# Case Report: Long-term response control in a patient with metastatic gastric squamous cell carcinoma treated with nivolumab and chemoradiotherapy

**DOI:** 10.3389/fimmu.2025.1552052

**Published:** 2025-08-07

**Authors:** Yu-ying Lei, Li Li, Ya-bin Shi, Xiao-hui Han, Yi-tong Li, Wen-jun Ren, Ai-xia Sui

**Affiliations:** ^1^ Department of Oncology, Hebei General Hospital, Shijiazhuang, Hebei, China; ^2^ Department of Pathology, Hebei General Hospital, Shijiazhuang, Hebei, China

**Keywords:** EBV-associated gastric squamous cell carcinoma, immunotherapy, long-term response, nivolumab, programmed cell death ligand-1 (PD-L1) positive

## Abstract

Primary gastric squamous cell carcinoma (GSCC) is rare, typically associated with poor survival rates and unsatisfactory outcomes from conventional treatments including surgery, radiotherapy, and chemotherapy. It remains unclear whether nivolumab is as effective for GSCC as it is for gastric adenocarcinoma. Herein, we present the case of a 66-year-old man diagnosed with Epstein-Barr virus-positive metastatic GSCC, involving the liver, multiple lymph nodes, and invasion into the spleen, pancreatic body and tail, and splenic vein. The patient received nivolumab with oxaliplatin, leucovorin, and fluorouracil plus local radiotherapy as first-line treatment; nivolumab, paclitaxel polymer micelles, and carboplatin as the second-line treatment, and nivolumab and apatinib as the third-line treatment. The patient responded remarkably (survival period for 49 months) with manageable treatment toxicity. Genomic and immune characteristics were analyzed to understand the underlying disease mechanisms. In conclusion, integrated therapy (immunotherapy, chemotherapy, local radiotherapy, and anti-angiogenic therapy) may be the ideal treatment approach for patients with GSCC with multiple metastases; however, prospective studies are needed to verify its efficacy.

## 1 Introduction

Primary gastric squamous cell carcinoma (GSCC) is an epithelial malignant tumor characterized by differentiated squamous cells. This rare tumor type accounts for approximately 0.04–0.5% of primary gastric cancers (GCs) (1, 2). GSCC-related symptoms, such as weight loss and abdominal discomfort, resemble those of gastric adenocarcinoma; however, patients with primary GSCC generally experience poorer survival outcomes (3). Surgical intervention combined with adjuvant radiotherapy and chemotherapy is currently the optimal treatment option (3, 4). Owing to limited published reports, there is no consensus on the standard therapeutic regimen for primary GSCC. Furthermore, the opportunity for radical surgery to prolong survival is usually lost as most patients are diagnosed at a late stage. Therefore, there is an urgent need to develop novel therapeutic strategies.

Immunotherapy appears promising for cancers, offering significant survival outcomes in select patients. Nivolumab is a programmed cell death protein-1 (PD-1) immune-regulating antibody. Programmed cell death ligand-1 (PD-L1) binding to PD-1 on cytotoxic T lymphocytes (CTLs) blocks their activation and trafficking to tumors, facilitating tumor’s evasion of immune surveillance (5). The anti-PD-1 or anti-PD-L1 antibodies can reactivate CTLs in cancers by disrupting the PD-1/PD-L1 axis, and enhancing anti-tumor activity across various solid tumors, such as gastric, colorectal, and lung cancers (6–9). Recent trials have shown encouraging effects of nivolumab in advanced gastric, gastroesophageal junction, and esophageal adenocarcinomas (10–12), however, its impact on GSCC has not been reported.

Patients with PD-L1-positive GC with high microsatellite instability and high tumor mutational burden (TMB) have demonstrated superior survival rates (11). Despite our patient being PD-L1-positive with microsatellite stability and a low TMB, prolonged survival was observed. Analyzing the genetic and immune profile differences could help to elucidate the mechanisms underlying a favorable prognosis. However, published immunotherapy trials have mostly focused on gastric adenocarcinoma, with limited reports on GSCC. Epstein-Barr virus-positive (EBV[+]) GC accounts for 9% of all GC cases (13). Several studies have indicated excellent remission rates in patients with EBV(+) GC following immunotherapy, suggesting EBV as a latent biomarker (14, 15). Xie et al. reported that the objective response rate in EBV(+) metastatic GC was 100% following combined immunotherapy (16).

Owing to its low incidence, the prognosis and treatment of GSCC remains uncertain. Here, we present the case of a 66-year-old man with metastatic GSCC who exhibited an outstanding response and manageable toxicity to nivolumab. Genomic and immune characteristics were analyzed to explore the underlying mechanisms.

## 2 Description

### 2.1 Diagnostic assessment

**Figure 1:**
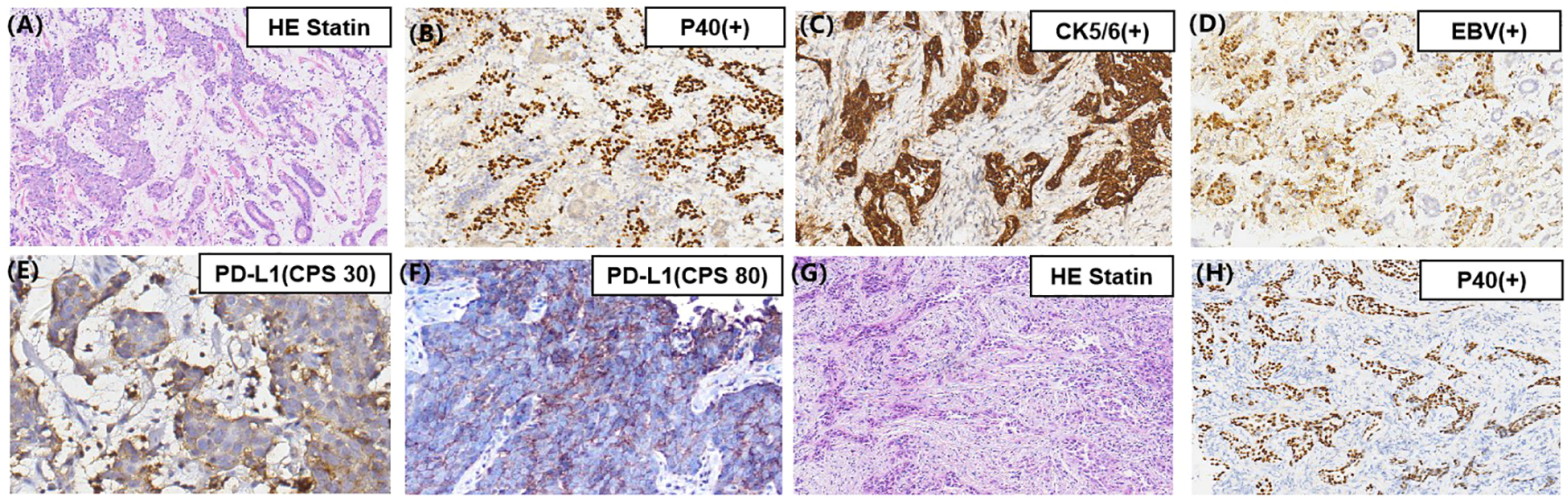
**(A)** Pathology of the gastric mass showing a poorly differentiated SCC; **(B)** Immunohistochemical staining showing tumor cells positive for P40 and **(C)** CK5/6; **(D)** The brown cells are the cells harboring EBV infection (EBV-encoded small RNA *in situ* hybridization, EBER-ISH ×200 magnification images); **(E)** Immunohistochemical staining indicating broadly positive programmed death-ligand 1 expression in the primary gastric mass; **(F)** Immunohistochemical staining indicating broadly positive programmed death-ligand 1 expression in the second biopsy of the gastric mass (original magnification, ×400); **(G)** Pathology of left subclavian lymph node showing a poorly differentiated SCC; **(H)** Immunohistochemical staining showing tumor cells of the left subclavian lymph node positive for P40 (original magnification, ×200).

**Figure 2:**
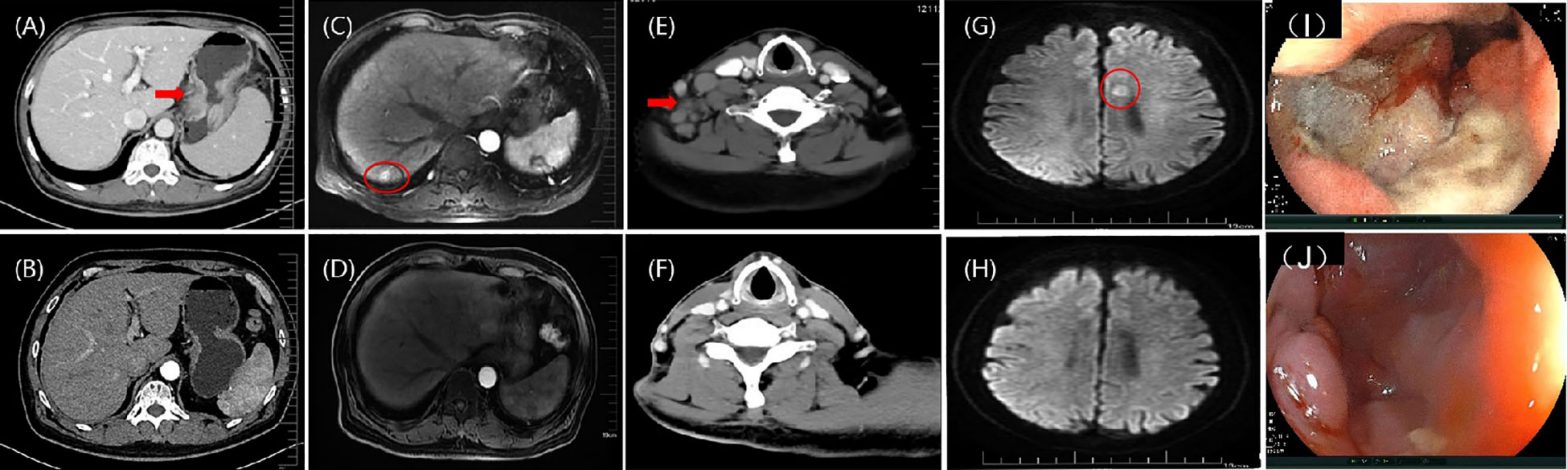
**(A)** According to an abdominal contrast-enhanced CT scan, there were a malignant gastric mass; **(B)** After eight cycles of nivolumab and FOLFOX, the contrast-enhanced CT image indicates that the gastric mass is PR; **(C)** According to hepatic artery phase MRI image, there was metastatic tumor in the liver; **(D)** Following eight cycles of FOLFOX + nivolumab, the hepatic artery phase MRI images indicating PR in the liver metastasis; **(E)** A cervical contrast-enhanced CT image showing multiple lymph nodes in the right supraclavicular region; **(F)** After local radiotherapy, the cervical contrast-enhanced CT image shows that the lymph node metastasis is PR; **(G)** The DWI sequence of MRI image showing a single metastasis in the left side of corpus callosum (11mm×8mm); **(H)** After six cycles of paclitaxel polymer micelles and carboplatin + nivolumab, the DWI sequence of MRI image shows that the brain metastasis is CR; **(I)** Endoscopic image of the stomach before treatment; **(J)** Endoscopic image of the stomach after treatment, indicates that the primary gastric cancer lesion is PR. CT, computed tomography; FOLFOX, leucovorin calcium (folinic acid), fluorouracil, and oxaliplatin; MRI, magnetic resonance imaging; DWI, diffusion weighted imaging; CR, complete response; PR, partial response.

A 66-year-old Chinese man presented at a local hospital in early September 2020 with stomach bloating, occasionally accompanied by nausea and vomiting. Gastroscopy revealed a poorly differentiated carcinoma (**Figures 1A**, **2I**). Tumor cells showed strong expression of P40, negative expression of Syn, CD56, CgA. The pathological analysis confirmed the presence of squamous cell carcinoma with an EBV(+) status (**Figures 1B–D**). Immunohistochemical staining data indicated high PD-L1 protein expression, with a combined positive score (CPS) of 30 (**Figure 1E**). Additionally, immunohistochemical analysis showed negative expression of human epidermal growth factor receptor 2 and overexpression of MSH2, MLH1, PMS2, and MSH6, indicating proficient mismatch repair. The patient had a history of smoking but no family history of malignancy. He visited our department at the end of September 2020. Physical examination revealed double clavicle fullness, hard and fixed lymph nodes approximately 2 ×2 cm in size in the right supraclavicular area, and no tenderness. His height and weight were 174 cm and 67 kg, respectively. An abdominal and pelvic computed tomography (CT) scan on September 28, 2020 showed significant, uneven thickening of the gastric fundus and body wall (**Figure 2A**). The possibility of adjacent spleen, pancreatic body/tail, or splenic vein invasion was considered. Multiple enlarged lymph nodes surrounding the lesion and in the retroperitoneal and mediastinal regions indicated metastasis. The occurrence of multiple liver metastases was also considered (**Figure 2C**). Ultrasonography of the bilateral supraclavicular and subclavian lymph nodes showed multiple enlarged lymph nodes in the left subclavian and right supraclavicular regions, further suggesting metastasis. Pathological examination of the left subclavian lymph node confirmed metastatic carcinoma, consistent with squamous cell carcinoma (**Figures 1G, H**).

Positron emission tomography (PET)-CT suggested GC with multiple lymph nodes metastases, potentially invading the spleen and pancreas. Metastases were detected in the right supraclavicular, left subclavian, and mediastinal lymph nodes and in the liver. In summary, the patient was diagnosed with poorly differentiated squamous cell carcinoma of the gastric fundus and body, with invasion of the spleen and pancreas (stage cT4bN3M1 IVB G3), along with hepatic and multiple lymph nodes metastases was diagnosed. The results of next-generation sequencing revealed four gene mutations (**Supplementary File 1**), microsatellite stability status, unknown TMB, and positive PD-L1 (22C3) protein expression with a CPS of 30.

### 2.2 Therapeutic intervention, follow-up, and outcomes

The patient demonstrated a strong desire to control the disease; he had a good Eastern Cooperative Oncology Group score and high PD-L1 expression. He was treated with the following protocol: oxaliplatin (85 mg), leucovorin (400 mg), and fluorouracil (400 mg) on day 1, followed by fluorouracil (200 mg) on days 1–2. Additionally, nivolumab (3mg/kg) was administered on day 1 and repeated every 2 weeks as first-line therapy. Enhanced CT imaging was conducted every 8 weeks to evaluate the treatment efficacy. After 8 weeks, there was noticeable thinning of the stomach wall, with the best response assessed as partial, based on Response Evaluation Criteria in Solid Tumors 1.1 criteria (**Figure 2B, D**). Subsequently, the patient continued treatment with leucovorin calcium (folinic acid), fluorouracil, and oxaliplatin (FOLFOX) combined with nivolumab for up to eight cycles, achieving a sustained partial response. Chemotherapy-related adverse effects included grade 1 nausea and vomiting, and grade 2 decreased neutrophil and white blood cell counts, with no immune-associated adverse reactions observed. The patient’s quality of life remained excellent. Based on this outstanding response to immunotherapy, nivolumab was continued every 2 weeks.

In October 2021, multiple lymph nodes in the right posterior cervical triangle and right supraclavicular region were found to be enlarged (**Figure 2E**). A lymph node biopsy was performed in the right supraclavicular region. Pathological examination revealed poorly differentiated squamous cell carcinoma. To exclude other possible primary squamous cell carcinomas, PET-CT, nasopharyngoscopy, and nasopharyngeal magnetic resonance imaging (MRI) were performed, revealing no additional primary tumors. Following a multidisciplinary team conference, the patient underwent external-beam radiotherapy for cervical lymph node metastases, receiving a total of 33 fractions (PGTVnd, 62 Gy/33f; PTV1, 59.40 Gy/33f; PTV2, 54.12 Gy/33f). Throughout radiotherapy, the patient continued receiving nivolumab as maintenance treatment for up to nine cycles. The tumor regressed gradually and continuously up to the most recent follow-up, culminating in a complete response evaluation (**Figure 2F**).

In May 2022, the patient developed limb convulsions. Brain MRI showed metastases in the left side of the corpus callosum (**Figure 2G**). PET-CT conducted on May 27, 2022, identified metastases in the 9th thoracic vertebrae, mediastinum between the thoracic aorta, and diaphragmatic posterior flexus of the stomach. Multiple para-aortic hypermetabolic lymph nodes, consistent with the manifestations of multiple lymph node metastasis, were discovered in the 9th thoracic vertebra and intrathoracic aorta compared with the previous PET-CT findings. Additional hypermetabolic lymph nodes were identified in the mediastinum than previously observed. The previous PET-CT had showed disappearance of the right supraclavicular hypermetabolic lymph nodes, and no significant changes in the remaining hypermetabolic lymph nodes. On May 31, 2022, a gastroscopy and a bite test was performed in accordance with the PET-CT findings. Gastroscopic pathology findings confirmed a poorly differentiated squamous cell carcinoma (**Figure 2J**). The next-generation sequencing revealed two gene mutations (**Supplementary file 2**), microsatellite stability status, a low TMB with 5.2 Muts/Mb, and positive PD-L1 (22C3) protein expression with a CPS of 80 (**Figure 1F**). The patient was treated with nivolumab (3 mg/kg) on day 1, then every 2 weeks. Combined paclitaxel polymer micelles (390 mg initially, then 450 mg for subsequent five cycles) and carboplatin (0.5 g) was administered tri-weekly as second-line therapy. Brain metastases progressively reduced, achieving complete response at the latest follow-up (**Figure 2H**). Other metastases demonstrated reduced or stable disease. The patient continued treatment with nivolumab until November 2022, maintaining favorable therapeutic effects.

**Figure 3:**
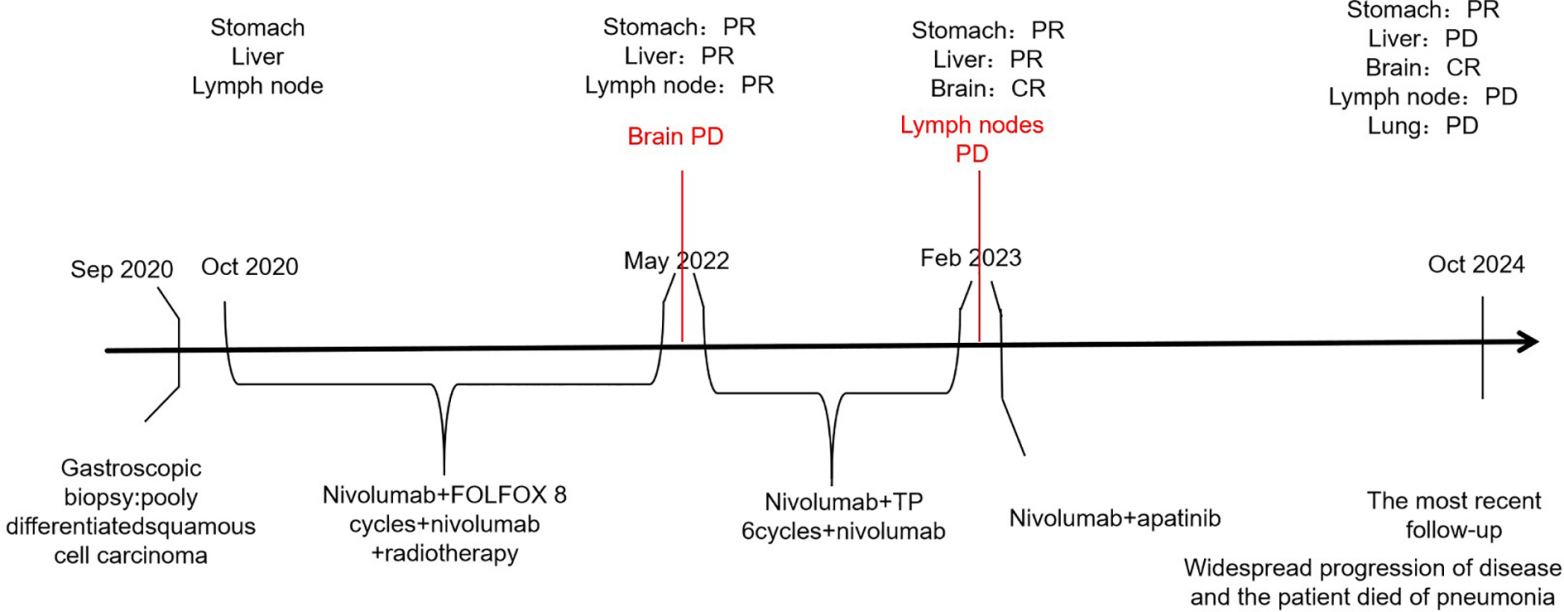
Timeline of the clinical course. FOLFOX, leucovorin calcium (folinic acid), fluorouracil, and oxaliplatin; TP, paclitaxel polymer micelles and carboplatin.

Subsequently, nivolumab administration was interrupted for 3 months, owing to coronavirus disease-2019. In February 2023, PET-CT showed enlargement of the mediastinal lymph node and left adrenal gland metastases, with stability observed in the other lesions. Following multidisciplinary team conferences, combination therapy with immune-targeting and anti-angiogenic agents was recommended. The patient received nivolumab (240 mg) on day 1, followed by twice-weekly administration, along with apatinib mesylate tablets (0.5 g daily) as third-line treatment. The tumor was evaluated and was shown to have reduced to the point of stable disease. However, in the most recent follow-up, the patient experienced widespread progression and ultimately succumbed to pneumonia. In conclusion, he achieved a survival duration of 49 months (**Figure 3**).

## 3 Discussion

GSCC is generally diagnosed at an advanced stage and is associated with poor survival outcomes (2, 3). However our case report presents a positive survival outcome. This is the first case report to demonstrate long-term tumor response control and the safety of combining nivolumab with chemoradiotherapy and anti-angiogenic therapy for metastatic GSCC.

During treatment, needle biopsies of the primary lesion and metastatic lymph nodes were performed, and the pathological results from all sites indicated squamous cell carcinoma, which supported a mono-causal origin. In addition, the metastasis pattern of gastric adenocarcinoma primarily involves the abdominal cavity (17); however, mediastinal lymph node metastasis was mainly observed in this case, which reflects the characteristics of GSCC invasion. This finding, indicative of GSCC, suggested that the prognosis would be poorer when compared with gastric adenocarcinoma (3).

In GSCC, the median overall survival is reported to be 8.9 months, and the 5-year overall survival is 32.7% (1, 3). However, our patient exhibited long remission duration (>43 months), suggesting prolonged survival. This outcome can be attributed to several factors. First, the good prognosis of this patient may have been attributed to the high expression of PD-L1 on the tumor cells. PD-L1 expression on cancer cells combined with PD-1 on CTLs facilitated tumor cell escape from antitumor immunity. The first CPS test score for PD-L1 (22C3) protein expression was 30, while the second score had increased to 80. High PD-L1 levels are associated with cancer progression (5). Therefore, anti-PD-1/PD-L1 therapy may have inhibited tumor immune escape, improving treatment efficacy. Second, the characteristics of EBV-related cancers may have contributed to their excellent responses. EBV(+) GCs have more tumor-infiltrating lymphocytes than EBV (–) GCs (18, 19). Therefore, the expression of PD-1 is higher in EBV(+) GCs than that in EBV (–) GCs (20). Patients with EBV(+) GCs benefit more from receiving PD-1 blockade treatment than those with EBV (–) GCs (21). Third, our patient had a mutation in the AT-rich interactive domain 1A (*ARID1A*) gene (22). Patients with *ARID1A* mutations have a better prognosis and benefit more from PD-1 inhibitor therapy compared with those with wild-type *ARID1A* tumors (22, 23). Frequent mutations in *ARID1A*, which encodes a member of the SWItch/sucrose non-fermentable chromatin-remodeling family, occur in 73% of patients with EBV(+) GCs cases (22). In contrast to our study, previous research has indicated that a HER-2-positive status may contribute to a better prognosis in patients with GC receiving immunotherapy (24, 25). Previous studies have shown that the co-expression of PD-L1 and HER2 could lead to GC immune escape, indicating that a HER-2-negative status was beneficial in improving immune efficacy (6). Fourth, the patient was treated with immunotherapy in combination with antiangiogenic agents as the final line of therapy. An increasing number of studies have suggested that angiogenesis occurs with immunosuppression (26). Accordingly, anti-angiogenesis therapy may have enhanced anti-tumor immune efficacy. Fifth, lymphocytes play a pivotal role in the immune system; therefore, we conducted an analysis of lymphocyte subsets during the treatment period. Following the initiation of immunotherapy in conjunction with chemotherapy, we observed an upward trend in total T lymphocytes, which suggests a favorable prognosis. However, this trend subsequently declined, indicating potential recurrence and metastasis of the tumor. In February 2023, PET-CT imaging revealed an increase and enlargement of metastatic lymph nodes (**Supplementary Figure 1**).

Surgery is the primary form of treatment and when combined with adjuvant therapy, it may greatly improve survival rates (3). The patient in this case did not undergo surgery because his disease was initially found at an advanced stage with liver and multiple lymph node metastases, along with invasion of the adjacent spleen, pancreatic body tail, and splenic vein. Surgery alone is not ideal for advanced cases. Therefore, additional treatment strategies are urgently needed. However, most results have been sub-optimal, and no standard therapy for GSCC has been defined (2, 15). Based on the adjuvant treatment regimen for esophageal squamous carcinoma and gastric adenocarcinoma, we chose nivolumab and FOLFOX as first-line treatment. A significant response, early tumor shrinkage, long-term response control, and controlled adverse reactions were observed. As the patient progressed, a second biopsy revealed squamous cell carcinoma. We chose paclitaxel polymer micelles and carboplatin as second-line treatment based on the adjuvant treatment for esophageal squamous carcinoma. In addition, multidisciplinary methods, such as nivolumab plus FOLFOX, radiotherapy (local therapy), and anti-angiogenic therapy, could be more appropriate therapeutic strategies for patients with multiple metastases. Thus, we report the first case of excellent clinical efficacy of nivolumab in GSCC, which has previously been reported in the treatment of many tumors (9, 10, 12, 27–29).

## 4 Conclusion

Here, we report a case of stage IV GSCC that achieved long-term response control through immunotherapy, chemotherapy, local radiotherapy, and anti-angiogenic therapy. Integrated therapy may be the ideal treatment for patients with GSCC and multiple metastases. However, further prospective studies are required to verify the efficacy of this treatment.

## Data Availability

The original contributions presented in the study are included in the article/**Supplementary Material**. Further inquiries can be directed to the corresponding author.
