# Supplementary figures and images for: Case Report: Long-term response control in a patient with metastatic gastric squamous cell carcinoma treated with nivolumab and chemoradiotherapy
Source: Front Immunol. 2025 Aug 7;16:1552052. doi: 10.3389/fimmu.2025.1552052 (PMC12367739; doi:10.3389/fimmu.2025.1552052)

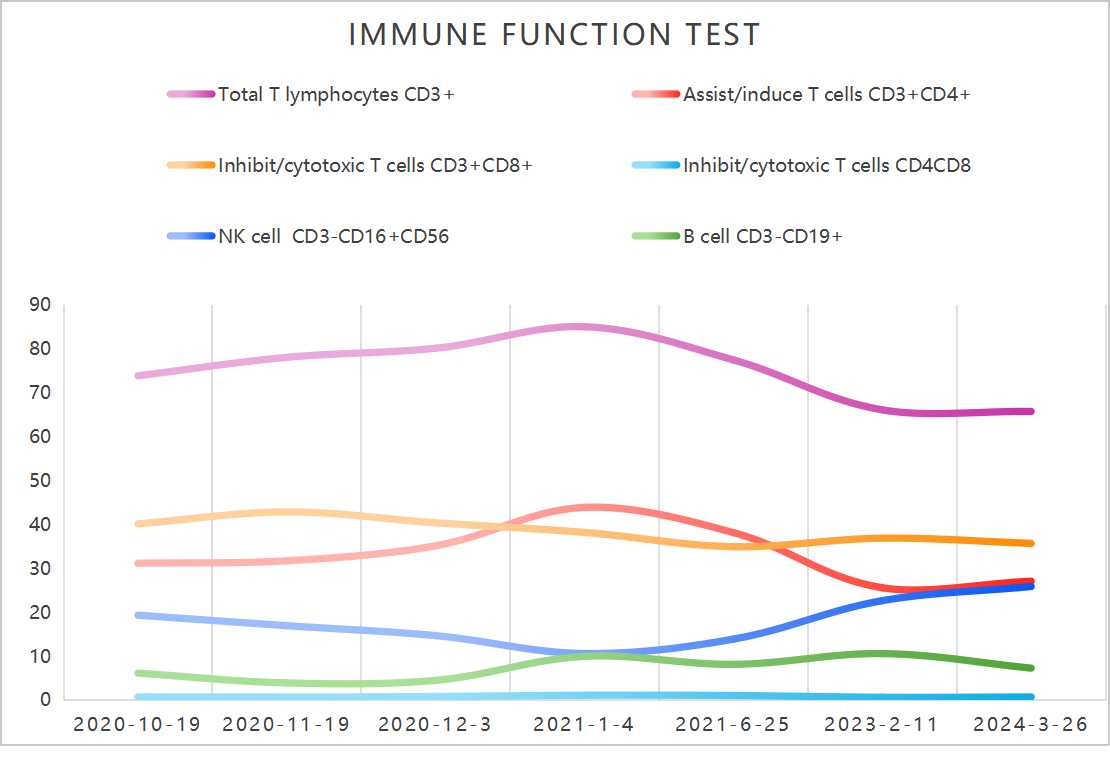

Supplement: Supplementary file 1 [file Image1.jpg]
