# Supplementary material for: Case Report: Long-term response control in a patient with metastatic gastric squamous cell carcinoma treated with nivolumab and chemoradiotherapy
Source: Front Immunol. 2025 Aug 7;16:1552052. doi: 10.3389/fimmu.2025.1552052 (PMC12367739; doi:10.3389/fimmu.2025.1552052)
Supplement: Supplementary file 2 [file Table1.doc]

**Supplementary file 1 |** The four gene mutations of the patient.

| **Gene** | **Mutations(amino acid change)** | **Mutations(nucleotide change)** | **Mutation abundance** |
| --- | --- | --- | --- |
| NRAS | G13D | 38G>A | 18.12% |
| EMSY | A1198V | 3593C>T | 53.07% |
| RAD51D | V66M | 196G>A | 39.59% |
| TET2 | 1N483D | 1447A>G | 38.03% |
