# Supplementary material for: Case Report: Long-term response control in a patient with metastatic gastric squamous cell carcinoma treated with nivolumab and chemoradiotherapy
Source: Front Immunol. 2025 Aug 7;16:1552052. doi: 10.3389/fimmu.2025.1552052 (PMC12367739; doi:10.3389/fimmu.2025.1552052)
Supplement: Supplementary file 3 [file Table2.doc]

**Supplementary file 2 |** The two gene mutations of the patient.

| **Gene** | **Mutations** | **Mutation abundance** |
| --- | --- | --- |
| PIK3CA | E542K exon10 | 8% |
| ARID1A | c.2732+1G>A | 8% |
